# Supplementary figures and images for: Test re-test reliability and construct validity of the star-track test of manual dexterity
Source: PeerJ. 2015 Apr 23;3:e917. doi: 10.7717/peerj.917 (PMC4411521; doi:10.7717/peerj.917)

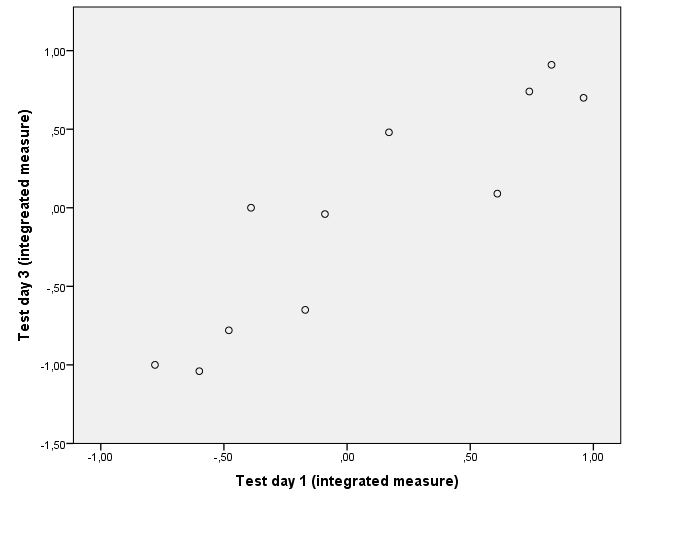

Supplement: Supplemental Information 1 [file peerj-03-917-s001.png]
