# Supplementary material for: Test re-test reliability and construct validity of the star-track test of manual dexterity
Source: PeerJ. 2015 Apr 23;3:e917. doi: 10.7717/peerj.917 (PMC4411521; doi:10.7717/peerj.917)
Supplement: Supplemental Information 2 [file peerj-03-917-s002.docx]

| Data for the construct validity study | | | | | | | | |
| --- | --- | --- | --- | --- | --- | --- | --- | --- |
|  | time (s) | rank | Difference from  middle rank | error | rank | Difference from  middle rank | Integrated  measure |  |
| Baseline |  |  |  |  |  |  |  | mean |
| 1 | 67 | 44,5 | 0,10 | 0 | 4 | -0,90 | -0,80 | -0,39321 |
| 2 | 58 | 28 | -0,31 | 1 | 14,5 | -0,64 | -0,95 |  |
| 3 | 63 | 34 | -0,16 | 2 | 26,5 | -0,35 | -0,51 |  |
| 4 | 83 | 71 | 0,75 | 0 | 4 | -0,90 | -0,15 |  |
| 5 | 48 | 12,5 | -0,69 | 3 | 40 | -0,01 | -0,70 |  |
| 6 | 37 | 2 | -0,95 | 13 | 78,5 | 0,94 | -0,01 |  |
| 7 | 70 | 52 | 0,28 | 3 | 40 | -0,01 | 0,27 |  |
| 8 | 80 | 67,5 | 0,67 | 1 | 14,5 | -0,64 | 0,02 |  |
| 9 | 61 | 33 | -0,19 | 1 | 14,5 | -0,64 | -0,83 |  |
| 10 | 83 | 71 | 0,75 | 0 | 4 | -0,90 | -0,15 |  |
| 11 | 60 | 31,5 | -0,22 | 2 | 26,5 | -0,35 | -0,57 |  |
| 12 | 64 | 35,5 | -0,12 | 5 | 57 | 0,41 | 0,28 |  |
| 13 | 48 | 12,5 | -0,69 | 6 | 64 | 0,58 | -0,11 |  |
| 14 | 46 | 8 | -0,80 | 3 | 40 | -0,01 | -0,81 |  |
| 15 | 80 | 67,5 | 0,67 | 3 | 40 | -0,01 | 0,65 |  |
| 16 | 53 | 20 | -0,51 | 4 | 50,5 | 0,25 | -0,26 |  |
| 17 | 43 | 4,5 | -0,89 | 2 | 26,5 | -0,35 | -1,23 |  |
| 18 | 50 | 15,5 | -0,62 | 1 | 14,5 | -0,64 | -1,26 |  |
| 19 | 65 | 38,5 | -0,05 | 1 | 14,5 | -0,64 | -0,69 |  |
| 20 | 70 | 52 | 0,28 | 2 | 26,5 | -0,35 | -0,06 |  |
| Intervention 1 |  |  |  |  |  |  |  |  |
| 1 | 71 | 56 | 0,38 | 0 | 4 | -0,90 | -0,52 | 0,083333 |
| 2 | 48 | 12,5 | -0,69 | 2 | 26,5 | -0,35 | -1,04 |  |
| 3 | 66 | 41,5 | 0,02 | 8 | 72,5 | 0,79 | 0,81 |  |
| 4 | 108 | 79 | 0,95 | 1 | 14,5 | -0,64 | 0,31 |  |
| 5 | 46 | 8 | -0,80 | 3 | 40 | -0,01 | -0,81 |  |
| 6 | 44 | 6 | -0,85 | 5 | 57 | 0,41 | -0,44 |  |
| 7 | 70 | 52 | 0,28 | 6 | 64 | 0,58 | 0,86 |  |
| 8 | 57 | 25 | -0,38 | 5 | 57 | 0,41 | 0,02 |  |
| 9 | 66 | 41,5 | 0,02 | 2 | 26,5 | -0,35 | -0,32 |  |
| 10 | 90 | 75,5 | 0,86 | 1 | 14,5 | -0,64 | 0,22 |  |
| 11 | 57 | 25 | -0,38 | 3 | 40 | -0,01 | -0,40 |  |
| 12 | 86 | 74 | 0,83 | 1 | 14,5 | -0,64 | 0,19 |  |
| 13 | 55 | 21,5 | -0,47 | 6 | 64 | 0,58 | 0,11 |  |
| 14 | 67 | 44,5 | 0,10 | 4 | 50,5 | 0,25 | 0,35 |  |
| 15 | 65 | 38,5 | -0,05 | 5 | 57 | 0,41 | 0,36 |  |
| 16 | 58 | 28 | -0,31 | 2 | 26,5 | -0,35 | -0,65 |  |
| 17 | 47 | 10 | -0,75 | 7 | 69 | 0,70 | -0,05 |  |
| 18 | 55 | 21,5 | -0,47 | 13 | 78,5 | 0,94 | 0,47 |  |
| 19 | 84 | 73 | 0,80 | 5 | 57 | 0,41 | 1,21 |  |
| 20 | 70 | 52 | 0,28 | 7 | 69 | 0,70 | 0,99 |  |
| Intervention 2 |  |  |  |  |  |  |  |  |
| 1 | 67 | 44,5 | 0,10 | 1 | 14,5 | -0,64 | -0,54 | 0,104321 |
| 2 | 68 | 48 | 0,19 | 3 | 40 | -0,01 | 0,17 |  |
| 3 | 79 | 65 | 0,60 | 0 | 4 | -0,90 | -0,30 |  |
| 4 | 117 | 80 | 0,98 | 1 | 14,5 | -0,64 | 0,33 |  |
| 5 | 57 | 25 | -0,38 | 3 | 40 | -0,01 | -0,40 |  |
| 6 | 35 | 1 | -0,98 | 20 | 80 | 0,98 | 0,00 |  |
| 7 | 60 | 31,5 | -0,22 | 3 | 40 | -0,01 | -0,23 |  |
| 8 | 58 | 28 | -0,31 | 3 | 40 | -0,01 | -0,32 |  |
| 9 | 78 | 62,5 | 0,54 | 1 | 14,5 | -0,64 | -0,10 |  |
| 10 | 101 | 77 | 0,90 | 0 | 4 | -0,90 | 0,00 |  |
| 11 | 83 | 71 | 0,75 | 2 | 26,5 | -0,35 | 0,41 |  |
| 12 | 71 | 56 | 0,38 | 2 | 26,5 | -0,35 | 0,04 |  |
| 13 | 41 | 3 | -0,93 | 7 | 69 | 0,70 | -0,22 |  |
| 14 | 52 | 18,5 | -0,54 | 11 | 76,5 | 0,89 | 0,35 |  |
| 15 | 75 | 59 | 0,46 | 3 | 40 | -0,01 | 0,44 |  |
| 16 | 65 | 38,5 | -0,05 | 6 | 64 | 0,58 | 0,53 |  |
| 17 | 48 | 12,5 | -0,69 | 7 | 69 | 0,70 | 0,01 |  |
| 18 | 77 | 60,5 | 0,49 | 3 | 40 | -0,01 | 0,48 |  |
| 19 | 73 | 58 | 0,43 | 3 | 40 | -0,01 | 0,42 |  |
| 20 | 79 | 65 | 0,60 | 5 | 57 | 0,41 | 1,01 |  |
| Intervention 3 |  |  |  |  |  |  |  |  |
| 1 | 82 | 69 | 0,70 | 7 | 69 | 0,70 | 1,41 | 0,205556 |
| 2 | 78 | 62,5 | 0,54 | 5 | 57 | 0,41 | 0,95 |  |
| 3 | 67 | 44,5 | 0,10 | 0 | 4 | -0,90 | -0,80 |  |
| 4 | 90 | 75,5 | 0,86 | 1 | 14,5 | -0,64 | 0,22 |  |
| 5 | 52 | 18,5 | -0,54 | 3 | 40 | -0,01 | -0,56 |  |
| 6 | 43 | 4,5 | -0,89 | 8 | 72,5 | 0,79 | -0,10 |  |
| 7 | 59 | 30 | -0,26 | 2 | 26,5 | -0,35 | -0,60 |  |
| 8 | 64 | 35,5 | -0,12 | 4 | 50,5 | 0,25 | 0,12 |  |
| 9 | 65 | 38,5 | -0,05 | 3 | 40 | -0,01 | -0,06 |  |
| 10 | 107 | 78 | 0,93 | 1 | 14,5 | -0,64 | 0,28 |  |
| 11 | 79 | 65 | 0,60 | 1 | 14,5 | -0,64 | -0,04 |  |
| 12 | 77 | 60,5 | 0,49 | 5 | 57 | 0,41 | 0,90 |  |
| 13 | 51 | 17 | -0,58 | 6 | 64 | 0,58 | 0,00 |  |
| 14 | 50 | 15,5 | -0,62 | 9 | 74,5 | 0,84 | 0,22 |  |
| 15 | 71 | 56 | 0,38 | 5 | 57 | 0,41 | 0,79 |  |
| 16 | 68 | 48 | 0,19 | 4 | 50,5 | 0,25 | 0,43 |  |
| 17 | 46 | 8 | -0,80 | 11 | 76,5 | 0,89 | 0,09 |  |
| 18 | 68 | 48 | 0,19 | 3 | 40 | -0,01 | 0,17 |  |
| 19 | 56 | 23 | -0,43 | 9 | 74,5 | 0,84 | 0,41 |  |
| 20 | 70 | 52 | 0,28 | 3 | 40 | -0,01 | 0,27 |  |

Test re-test data

| Test re-test day 1 | Time (s) | rank | Difference from  middle rank | error | rank | Difference from  middle rank | Integrated measure | Mean |
| --- | --- | --- | --- | --- | --- | --- | --- | --- |
|  |  |  |  |  |  |  |  |  |
| 1 | 70 | 19 | 0,65 | 3 | 12,5 | 0,09 | 0,74 | 0,071146 |
| 2 | 42 | 2 | -0,83 | 9 | 20 | 0,74 | -0,09 |  |
| 3 | 92 | 22 | 0,91 | 0 | 3 | -0,74 | 0,17 |  |
| 4 | 48 | 8,5 | -0,26 | 11 | 21,5 | 0,87 | 0,61 |  |
| 5 | 49 | 10 | -0,13 | 1 | 7,5 | -0,35 | -0,48 |  |
| 6 | 69 | 18 | 0,57 | 5 | 16 | 0,39 | 0,96 |  |
| 7 | 64 | 15,5 | 0,35 | 0 | 3 | -0,74 | -0,39 |  |
| 8 | 47 | 6,5 | -0,43 | 1 | 7,5 | -0,35 | -0,78 |  |
| 9 | 48 | 8,5 | -0,26 | 3 | 12,5 | 0,09 | -0,17 |  |
| 10 | 62 | 14 | 0,22 | 8 | 18,5 | 0,61 | 0,83 |  |
| 11 | 61 | 13 | 0,13 | 0 | 3 | -0,74 | -0,61 |  |
|  |  |  |  |  |  |  |  |  |
|  |  |  |  |  |  |  |  |  |
| Test re-test day 3 |  |  |  |  |  |  |  |  |
|  | Time (s) | rank | Difference from mean | error | rank | Difference from mean | Integrated measure |  |
|  |  |  |  |  |  |  |  |  |
| 1 | 64 | 15,5 | 0,35 | 5 | 16 | 0,39 | 0,74 | -0,07115 |
| 2 | 47 | 6,5 | -0,43 | 5 | 16 | 0,39 | -0,04 |  |
| 3 | 82 | 21 | 0,83 | 1 | 7,5 | -0,35 | 0,48 |  |
| 4 | 45 | 3,5 | -0,70 | 8 | 18,5 | 0,61 | -0,09 |  |
| 5 | 57 | 11 | -0,04 | 0 | 3 | -0,74 | -0,78 |  |
| 6 | 66 | 17 | 0,48 | 4 | 14 | 0,22 | 0,70 |  |
| 7 | 73 | 20 | 0,74 | 0 | 3 | -0,74 | 0,00 |  |
| 8 | 41 | 1 | -0,91 | 2 | 10,5 | -0,09 | -1,00 |  |
| 9 | 46 | 5 | -0,57 | 2 | 10,5 | -0,09 | -0,65 |  |
| 10 | 59 | 12 | 0,04 | 11 | 21,5 | 0,87 | 0,91 |  |
| 11 | 45 | 3,5 | -0,70 | 1 | 7,5 | -0,35 | -1,04 |  |
